# Richard “Dicky” Dickinson (1669–1739) – satirical prints and probable acromegaly

**DOI:** 10.1007/s40618-026-02834-2

**Published:** 2026-02-13

**Authors:** Wouter W. de Herder

**Affiliations:** Erasmus MC, Department of Internal Medicine, Sector of Endocrinology, Rg520, Dr. Molewaterplein 40, Rotterdam, 3015 GD The Netherlands

**Fig. 1:**
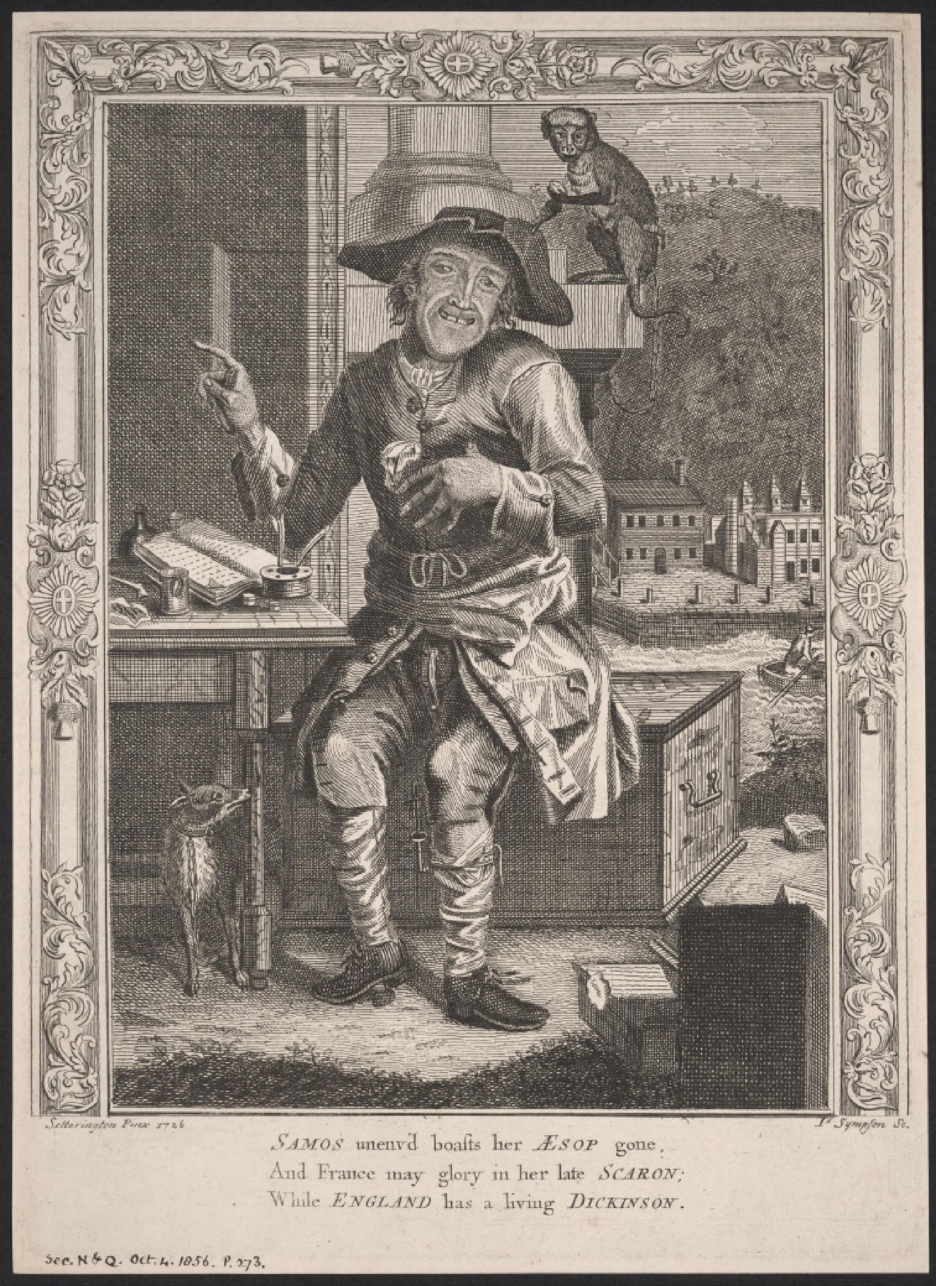
Satirical portrait of: Richard Dickinson. Print made by Joseph Sympson I after John Setterington (c.1726) © The British Museum, London, United Kingdom, 1868,0808.3512. Picture licensed to the author by The British Museum

**Fig. 2:**
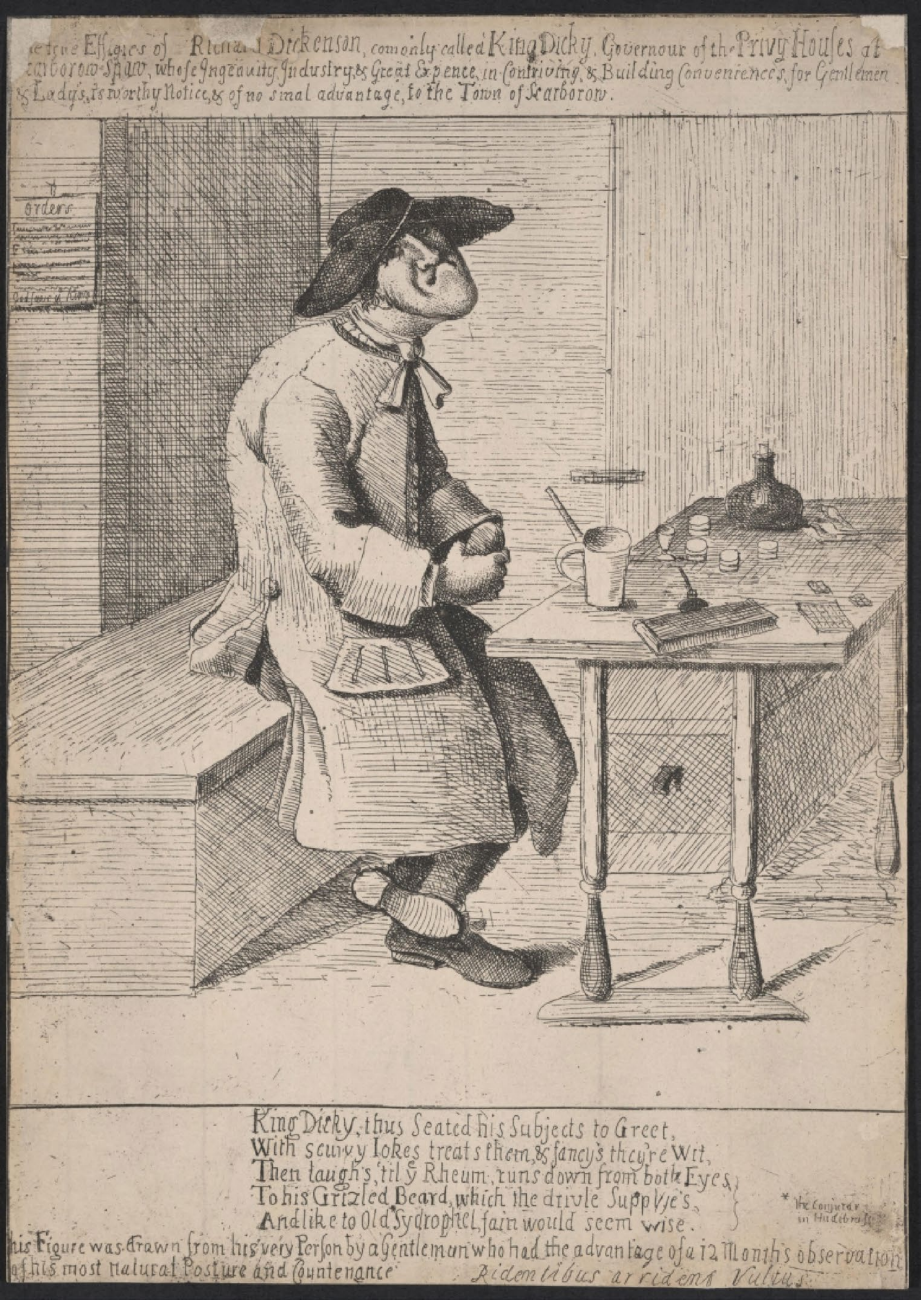
The true Effigies of Richard Dickinson. Anonymous (c.1725) © The British Museum, London, United Kingdom, 1851,0308.210. Picture licensed to the author by The British Museum

Richard “Dicky” Dickinson lived from his birth in 1669 to his death in 1739 in Scarborough, England. He became famous as a “*remarkable person*” in the 18th century and became the subject of at least 9 different, mostly satirical - portraits and engravings, often depicting him with his “*unusual features*” and sometimes accompanied by his pet fox and monkey. Several of these portraits/engravings are now held by the National Portrait Gallery and the British Museum in London, UK. (Figures 1 and 2).

Richard Dickinson became the First Governor of the Scarborough Spa, where he managed the original “Spa House”, where visitors came to drink curative mineral waters and he was responsible for collecting subscriptions from patrons, maintaining order, and providing basic amenities. He also oversaw the bottling and distant sale of spa water. A massive landslide in 1737 buried the Spa House and wells and Richard Dickinson died just two years later. He was famous for his sharp wit and eccentric behavior, sometimes imagining himself to be a king.

Dr. Leonard Portal Mark (1855–1930), a “Draughtsman of Pathology” (= medical illustrator) at St. Bartholomew’s Hospital, London, UK was diagnosed with acromegaly in 1905 at the age of 50. In 1912 he wrote his book: “Acromegaly - A personal Experience”, when he already had acromegaly for over 30 years [1, 2]. In 1914 he published a paper in The Lancet when his attention was drawn to the fact that signs and symptoms of acromegaly were to be found depicted in the portraits of Richard “Dicky” Dickinson [3]. In most of the prints, a very large lower jaw is depicted accompanied by lengthening of the face and pronounced nasolabial folds. The chest is massive with broad shoulders and the neck is “buried” between them. Most pictures show very large hands and feet. Dr. Leonard Mark concluded that Richard “Dicky” Dickinson must have been suffering from acromegaly and since that time descriptions of Richard “Dicky” Dickinson usually mention that he “*seems to have suffered*” from this disorder [3].
